# *Staphylococcus aureus subsp. anaerobius* strain ST1464 genome sequence

**DOI:** 10.4056/sigs.3748294

**Published:** 2013-10-15

**Authors:** Haitham Elbir, Catherine Robert, Ti Thien Nguyen, Grégory Gimenez, Sulieman M. El Sanousi, Jan-Ingmar Flock, Didier Raoult, Michel Drancourt

**Affiliations:** 1 Aix Marseille Université, URMITE, Marseille, France; 2 Faculty of Veterinary Medicine, University of Khartoum, Sudan; 3 Department of Microbiology, Tumor and Cell Biology, Stockholm, Sweden

**Keywords:** *Staphylococcus aureus* subsp. *anaerobius*, genome, SOLiD, Morel’s disease

## Abstract

*Staphylococcus aureus subsp. anaerobius* is responsible for Morel's disease in animals and a cause of abscess in humans. It is characterized by a microaerophilic growth, contrary to the other strains of *S. aureus*. The 2,604,446-bp genome (32.7% GC content) of *S. anaerobius* ST1464 comprises one chromosome and no plasmids. The chromosome contains 2,660 open reading frames (ORFs), 49 tRNAs and three complete rRNAs, forming one complete operon. The size of ORFs ranges between 100 to 4,600 bp except for two ORFs of 6,417 and 7,173 bp encoding segregation ATPase and non-ribosomal peptide synthase, respectively. The chromosome harbors *Staphylococcus* phage 2638A genome and incomplete *Staphylococcus* phage genome PT1028, but no detectable CRISPRS. The antibiotic resistance gene for tetracycline was found although *Staphylococcus aureus subsp. anaerobius* is susceptible to tetracycline *in-vitro*. Intact oxygen detoxification genes encode superoxide dismutase and cytochrome quinol oxidase whereas the catalase gene is impaired by a stop codon. Based on the genome, *in-silico* multilocus sequence typing indicates that *S. aureus subsp. anaerobius* emerged as a clone separated from all other *S. aureus* strains, illustrating host-adaptation linked to missing functions. Availability of *S. aureus subsp. anaerobius* genome could prompt the development of post-genomic tools for its rapid discrimination from *S. aureus*.

## Introduction

*Staphylococcus aureus subsp. anaerobius* (here referred as *S. aureus subsp. anaerobius*) is a Gram positive bacterium of veterinary interest and is responsible for Morel's disease, which is characterized by chronic subcutaneous abscesses near superficial lymph nodes in sheep and goat [1]. Morel's disease was described in Sudan, Saudi Arabia, Hungary, Spain, Denmark, Italy and Poland [1-7]. The only report of human infection was a case of septicemia in an Australian patient [8]. The causative agent of the disease is clonal and most of Morel's disease cases in the world are due to oxacillin-susceptible *S. aureus subsp. anaerobius* sequence type ST1464 [6]. The disease remains neglected and is rarely investigated in laboratory. Accordingly, the availability of the *S. aureus subsp. anaerobius* genome sequence may facilitate the development of molecular tools to improve the diagnosis and characterization of Morel's disease. Here we present a description of the complete genome sequence of *S. aureus subsp. anaerobius* strain ST1464 and its annotation, as well as a preliminary comparative analysis with the *S. aureus* subsp. *aureus* genome.

## Classification and features

**Figure 1:**
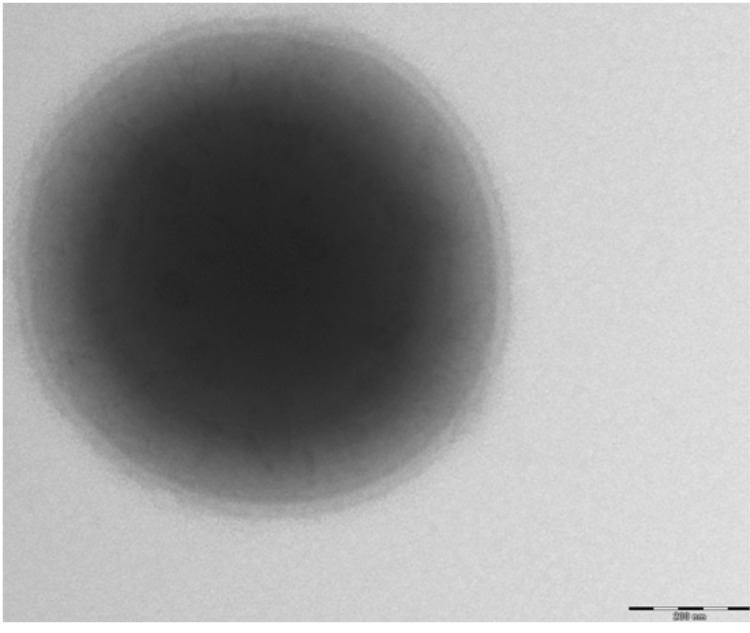
Transmission electron microscopy of *S. aureus subsp. anaerobius* strain st1464, using a Morgani 268D (Philips) at an operating voltage of 60kV. The scale bar represents 900 nm.

The *S. aureus subsp. anaerobius* strain ST1464 sequenced in this study was isolated from an abscess in the prescapular region of a sheep with Morel's disease in Khartoum state, Sudan [6]. *S. aureus subsp. anaerobius* is a Gram-positive, coccus-shaped bacterium (Figure 1 and Table 1) growing at 37 °C in a microaerophilic atmosphere containing < 8% oxygen.

**Table 1:** Classification and general features of *S. aureus subsp anaerobius* strain ST1464 according to the MIGS recommendations [9].

**MIGS ID**	**Property**	Term	**Evidence code^a^**
	Current classification	Domain *Bacteria*	TAS [10]
		Phylum *Firmicutes*	TAS [11-13]
		Class *Bacilli*	TAS [14,15]
		Order *Bacillales*	TAS [16,17]
		Family *Staphylococcaceae*	TAS [18,19]
		Genus *Staphylococcus*	TAS [16,20-22]
		Species *Staphylococcus aureus subsp. anaerobius*	TAS [1]
		Strain ST1464	TAS [7]
	Gram stain	Positive	TAS [23]
	Cell shape	Coccus	TAS [23]
	Motility	Nonmotile	TAS [23]
	Sporulation	Nonsporulating	TAS [23]
	Temperature range	30-40°C	TAS [23]
	Optimum temperature	37°C	TAS [1]
MIGS-6.3	Salinity	Tolerates 10% NaCl	TAS [1]
MIGS-22	Oxygen requirement	Microaerophilic	TAS [1]
	Carbon source	Fructose, sucrose	NAS
	Energy source	Fructose, sucrose	NAS
MIGS-6	Habitat	Subcutaneous abscess	TAS [1]
MIGS-15	Biotic relationship	free living	NAS
MIGS-14	Pathogenicity	Yes	NAS
	Biosafety level	2	NAS
	Isolation	Sheep abscess	TAS [1]
MIGS-4	Geographic location	Sudan	TAS [1]
MIGS-5	Sample collection time	September 2005	IDA
MIGS-4.1	Latitude	15.656' N	IDA
MIGS-4.1	Longitude	32.548' E	IDA
MIGS-4.3	Depth	Surface	IDA
MIGS-4.4	Altitude	382 m above sea level	IDA

**^a^**Evidence codes - IDA: Inferred from Direct Assay; TAS: Traceable Author Statement (i.e., a direct report exists in the literature); NAS: Non-traceable Author Statement (i.e., not directly observed for the living, isolated sample, but based on a generally accepted property for the species, or anecdotal evidence). These evidence codes are from the Gene Ontology project [24].

**Figure 2:**
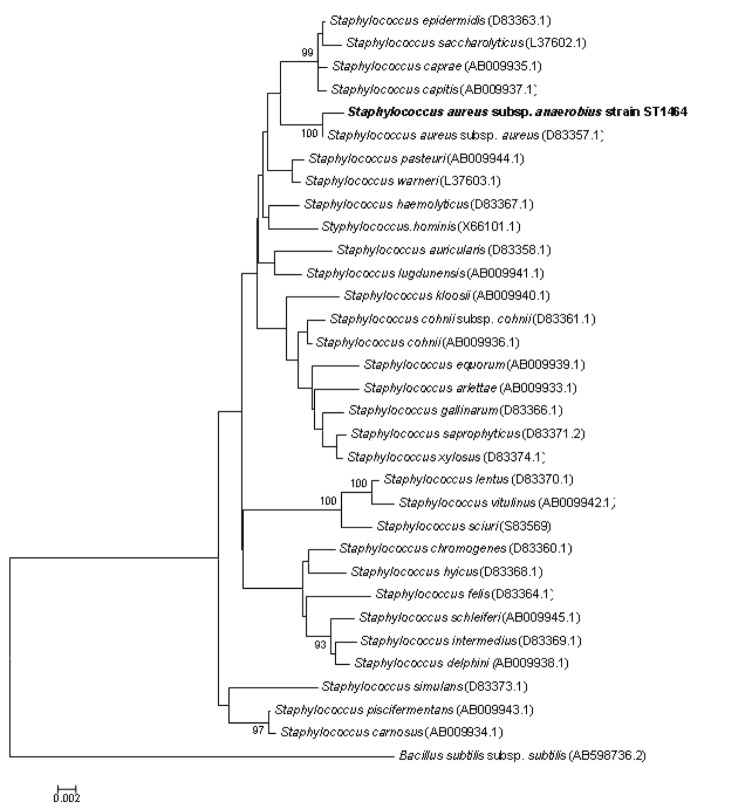
Phylogenetic tree depicting the relationship between *Staphylococcus aureus subsp. anaerobius* and other members of the genus *Staphylococcus* based on 1,311 base pairs of the 16S rRNA gene sequence aligned in Muscle. The tree was constructed by using the Neighbor-Joining method and Kimura 2-parameter model using MEGA5 software [26] and rooted with *Bacillus subtilis subsp. subtilis.* Bootstrap consensus trees were inferred from 100 replicates, only bootstrap values > 90% were indicated.

Biochemical features include positive tests for tube coagulase and DNase, negative tests for catalase, citrate, urease and ornithine decarboxylase. Using commercial Pheneplate system (PhPlate Microplate Techniques AB, Stockholm, Sweden) [25], positive reactions were obtained for fructose, sucrose and weak reaction for mannose, inosine and ribose. Negative reaction were observed for mannonic acid lacton L-arabinose, D- xylose, galactose, maltose, cellobiose, trehalose, palatinose, lactose, melibiose, lactulose, gentobiose, melezitose, raffinose, adonitol, D-arabitol, glycerol, maltitol, sorbitol, dulcitol, sorbose, deoxy-glucose, deoxy-ribose, rhamnose, D-fucose, L-fucose, tagatose, amygdalin, arbutin, keto-gluconate, gluconate, melbionate, galacturonic lacton, salicine, fumarate, malinate, malonate, pyruvate, tartarate, mannitol and xylitol. The type strain is deposited in the German Collection of Microorganisms and Cell Culture (DSMZ) as DSM 20714. The ST1464 strain exhibited a 99% nucleotide sequence similarity with the *Staphylococcus aureus* 16S rRNA gene (Genbank accession number D83357.1) (Figure 2).

**Figure 3:**
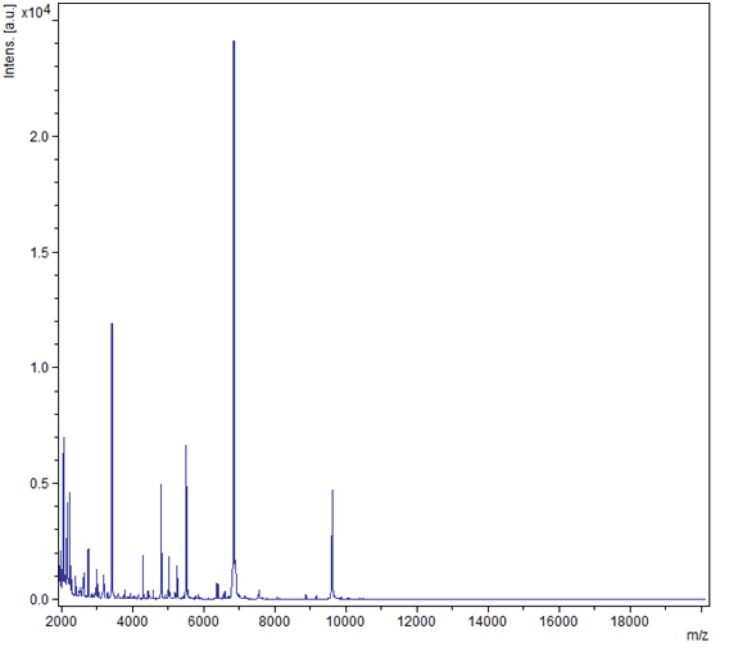
Reference mass spectrum from *S. aureus subsp. anaerobius* strain ST1464. Spectra from 4 individual colonies were compared and a reference spectrum was generated.

Matrix-assisted laser desorption/ionization time-of-flight mass spectrometry (MALDI-TOF-MS) (Brüker Daltonics, Bremen, Germany) was used as previously described [27]. Briefly, a pipette tip was used to pick one *S. aureus subsp. anaerobius* colony from a 5% blood agar plate and to spread it on a MTP 384 MALDI-TOF target plate (Brüker Daltonics). Smears were overlaid with 1.5 μL of matrix solution (saturated solution of alpha-cyano-4-hydroxycinnamic acid) in 50% acetonitrile, 2.5% tri-fluoracetic-acid and allowed to dry. MALDI-TOF without bacteria was used as a negative control and the positive control consisted of 1.5 μL of Brüker Bacterial Test Standard, a protein extract of *Escherichia coli* DH5alpha. Negative control spots remained negative and the positive control spots were identified as *E. coli* with score > 2, however, the S. aureus subsp. anaerobius spots yielded a score of 2.1 with the reference spectra of *S. aureus subsp. anaerobius* (Figure 3).

## Genome sequencing and annotation

### Genome project history

The organism was selected for sequencing on the basis of its economic importance in animal trade and public health. This Whole Genome Shotgun project has been deposited at DDBJ/EMBL/GenBank under the accession ANIT00000000. The version described in this paper is the first version, ANIT01000000. It consists of 100 large contigs. Table 2 shows the project information.

**Table 2:** Genome sequencing project information

**MIGS ID**	**Property**	**Term**
MIGS-31	Finishing quality	High-quality draft
MIGS-28	Libraries used	One 454 paired end 3-kb library
MIGS-29	Sequencing platforms	454 GS FLX Titanium
MIGS-31.2	Sequencing	24×
MIGS-30	Assemblers	Newbler version 2.5.3
MIGS-32	Gene calling method	Prodigal
	INSDC ID	PRJNA178987
	Genbank ID	ANIT00000000
	Genbank Date of Release	March 3, 2013
	Gold ID	Gi21982
MIGS-13	Project relevance	Vaccine development

### Growth conditions and DNA isolation

*S. aureus subsp. anaerobius* was grown in microaerophilic atmosphere on 5% sheep blood-enriched Columbia agar (bioMérieux, Marcy l’Etoile, France) at 37°C. Two hundred microliters of bacterial suspension were diluted into 1mL Tris EDTA buffer and incubated with lysozyme for 30 minutes at 37°C followed by an overnight incubation with Proteinase K at 37°C. DNA was purified by three successive phenol-chloroform extractions and ethanol precipitation at -20°C overnight. After centrifugation, the DNA was resuspended in 52 µL TE buffer. DNA concentration was measured by the Quant-it Picogreen kit (Invitrogen Saint Aubin, France) on the Genios_Tecan fluorometer at 60 ng/µL.

### Genome sequencing and assembly

Mechanical fragmentation of three µg of DNA was done on the Covaris device (KBioScience-LGC Genomics, Teddington, UK) using miniTUBE-Red 5Kb. DNA fragmentation was visualized using an Agilent 2100 BioAnalyzer on a DNA labchip 7500 with an optimal size of 3.2 kb. The library was constructed according to the 454 Titanium paired end protocol (Roche Applied Science, Mannheim, Germany). Circularization and nebulization generated a pattern with an optimum at 5,72 bp. After PCR amplification through 17 cycles followed by double size selection, the single stranded paired end library was quantified on the Quant-it Ribogreen kit (Invitrogen, Saint Aubin, France) on a Genios Tecan fluorometer at 1620 pg/µL. The library concentration equivalence was calculated as 2.61E+09 molecules/µL. The library was stocked at -20°C until used. The library was clonally amplified with 0.5 cpb and 1 cpb in two emPCR reactions respectively with the GS Titanium SV emPCR Kit (Lib-L) v2 (Roche). Yields of the emPCR were 10.76% and 14.04% for each semPCR condition. A total of 790,000 beads were loaded on the GS Titanium PicoTiterPlate PTP Kit 70×75 and sequenced with the GS Titanium Sequencing Kit XLR70 (Roche Applied Science, Mannheim, Germany). The run was performed overnight and then analyzed on the cluster through the gsRunBrowser and gsAssembler (Roche). A total of 230,000 passed filter wells were obtained and generated 80.57 Mb with an average 350-bp length. The passed-filter sequences were assembled on the gsAssembler from Roche with 90% identity and 40-bp overlap. Assembly yielded six scaffolds and 100 large contigs (> 500-bp), generating 24 × genome equivalents of a 2.6 Mb-genome.

### Genome annotation

The prodigal program was used to predict open reading frames (ORFs) from the 100 large contigs [28]. tRNAs were predicted using the Aragorn program [29] and rRNAs were predicted using RNAmmer. The predicted genes were Blasted against the non-redundant database. The functional annotation of predicted ORFs was performed using RPS-BLAST [30] against the cluster of orthologous groups (COG) database [31] and Pfam database [32]. TMHMM program was used for gene prediction with transmembrane helices [33] and signalP program was used for prediction of genes with peptide signals [34]. PHAST software was used for bacteriophage detection [35]. To estimate the similarity at the genome level between *S. aureus subsp. anaerobius* strain ST1464 and *S. aureus,* BLASTP was performed for genes with query coverage ≥70% and identity ≥30.

## Genome properties

**Figure 4:**
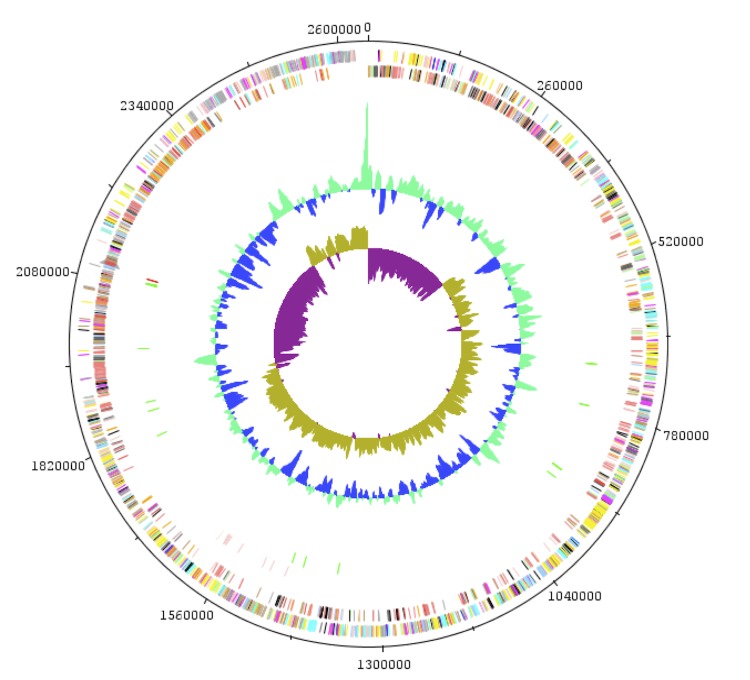
Graphical circular map of the chromosome. From outside to the center: Genes on the forward strand (colored by COG categories), genes on the reverse strand colored by COG categories), RNA genes (tRNAs green, rRNAs red), GC content, and GC skew.

**Figure 5:**
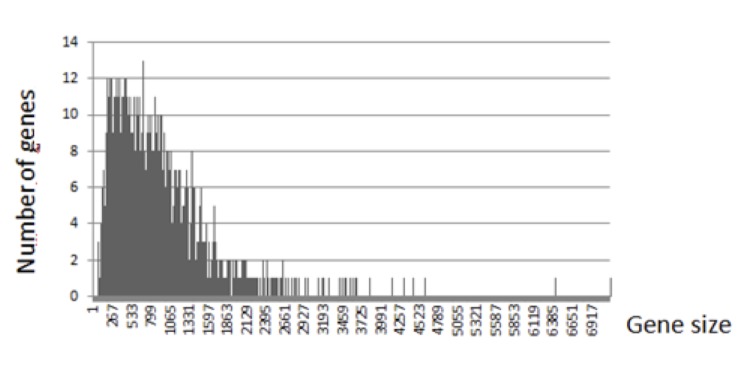
Graphical distribution of ORF size in the chromosome.

The genome consists of one circular 2,604,446-bp chromosome without a plasmid with a 32.7% G+C content. It comprises 2,660 ORFs, 49 tRNAs and three complete rRNAs. A total of 2,120 genes (78.17%) were assigned a putative function. The distribution of genes into COGs functional categories is presented in Table 3 and figure 4. The properties and the statistics of the genome are summarized in Table 4. ORF sizes ranged between 100 to 4,600 bp except for a 6,417-bp chromosome segregation ATPase gene and a 7,173-bp non-ribosomal peptide synthetase gene (Figure 5).

**Table 3:** Number of genes associated with the 25 general COG functional categories

**Code**	**Value**	**% age^a^**	**Description**
J	147	5.53	Translation, ribosomal structure and biogenesis
A	0	0	RNA processing and modification
K	185	6.95	Transcription
L	115	4.32	Replication, recombination repair
B	2	0.08	Chromatin structure dynamics
D	23	0.86	Cell cycle control, mitosis meiosis
Y	0	0	Nuclear structure
V	59	2.22	Defense mechanisms
T	63	2.37	Signal transduction mechanisms
M	116	4.36	Cell wall/membrane biogenesis
N	8	0.30	Cell motility
Z	0	0	Cytoskeleton
W	0	0	Extracellular structures
U	29	1.09	Intracellular trafficking secretion
O	78	2.93	Posttranslational modification, protein turnover, chaperones
C	120	4.51	Energy production conversion
G	172	6.47	Carbohydrate transport metabolism
E	227	8.53	Amino acid transport metabolism
F	74	2.78	Nucleotide transport metabolism
H	108	4.06	Coenzyme transport metabolism
I	67	2.52	Lipid transport metabolism
P	192	7.22	Inorganic ion transport metabolism
Q	43	1.62	Secondary metabolites biosynthesis, transport catabolism
R	337	12.67	General function prediction only
S	229	8.61	Function unknown
-	266	10	Not in COGs

^a^The total is based on the total number of protein coding genes in the annotated genome.

**Table 4:** Genome statistics

**Attribute**	**Value**	**% of total^a^**
Genome size (bp)	2,604,446	100
DNA coding region (bp)	2,154,549	82.72
DNA G+C content (bp)	8,510,011	32.7
Total genes	2,712	100
RNA genes	52	1.9
Protein-coding genes	2,660	98.08
Genes assigned to COGs	2,120	78.17
Genes assigned to pfam	2,148	79.2
Genes with peptide signals	146	5.38
Genes with transmembrane helices	680	25.07

^a^ The total is based on either the size of the genome in base pairs or the total number of protein coding genes in the annotated genome.

*S. aureus subsp. anaerobius* encodes genes related to oxidative stress protection, including two intact superoxide dismutase genes and intact cytochrome quinol oxidase genes, which mediate oxidative metabolism [36]. Contrary to *S. aureus*, *S. aureus subsp. anaerobius* encodes an impaired catalase gene [37]. *S. aureus subsp. anaerobius* genome contains one intact *Staphylococcus* phage 2638A [38] and one incomplete *Staphylococcus* phage PT1028 [38] but no detectable CRISPERs. No bacteriophage was detected by electron microscopic visualization of 100 fields. A tetracycline resistance gene was found, although the strain is susceptible to tetracycline *in-vitro*.

## Comparative genomics

**Figure 6:**
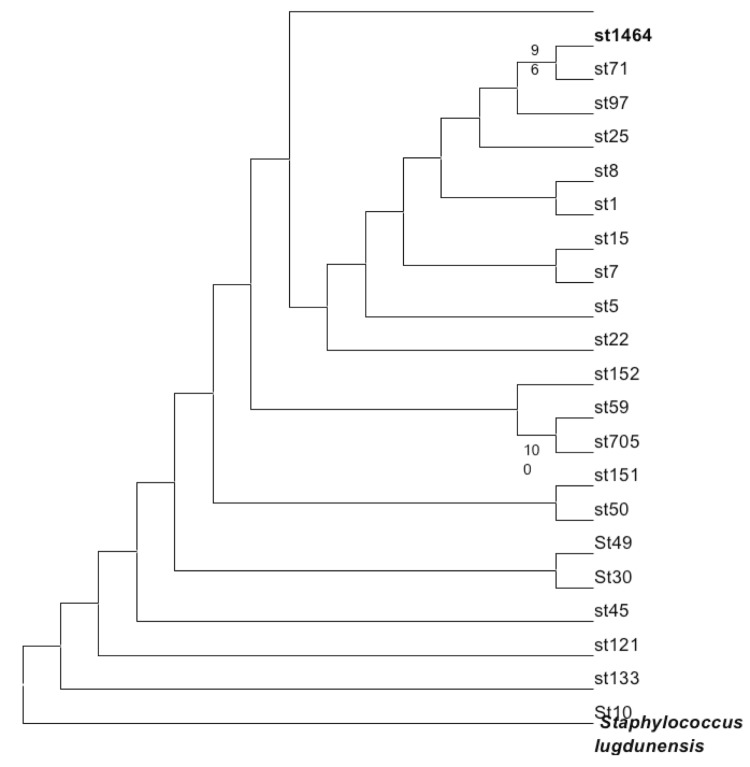
Maximum likelihood tree based on *in-silico* multilocus sequence typing of six genes (Acetyl-CoA acetyltransferase, putative glycerol uptake facilitator protein, shikimate 5-dehydrogenase, guanylate kinase, triosephosphate isomerase and putative phosphate acetyltransferase). Derived sequence types (St) are indicated at the end of each branch. It shows the relationship of *S. aureus subsp. anaerobius* with other *S. aureus* (ST 1464) and *Staphylococcus lugdunensis* as an external root. Only bootstrap values ≥90% were indicated at nodes.

The genome of *S. aureus* strains ranged between 2.67 to 3 Mb and contains between zero and three plasmids. Average pairwise amino acid sequence identity of 98% and partial colinearity were observed between *S. aureus* and *S. aureus subsp. anaerobius* chromosomes. Reads of *S. aureus subsp. anaerobius* were not mapped against all *S. aureus* plasmids. *S. aureus subsp. anaerobius* strain ST1464 contains *S. aureus* virulence genes including *cna* collagen binding protein, *ica*R *ica* operon transcriptional regulator, *ica*A intercellular adhesion protein A, *ica*D intercellular adhesion protein D, *ica*B intercellular adhesion protein B, *ica*C intercellular adhesion protein C, *aur* zinc metalloproteinase aureolysin gene, *geh* glycerol ester hydrolase gene, *isd*B conserved hypothetical protein, *hys*A hyaluronate lyase precursor, *sdr*C beta-neurexin binding protein gene, *eta* exfoliative toxin A and *sea* staphylococcal enterotoxin A precursor gene. However, it lacks genes encoding the serine protease, the clumping factor CIFA, enterotoxin B, adenosine synthase A and toxic shock syndrome toxin, panton-valentine toxin and staphylokinase SAK. The extracellular adherence protein Eap gene is interrupted by a stop codon. Based on its genome, an *in-silico* multilocus sequence typing (Figure 6) indicates that *S. aureus subsp. anaerobius* emerged from *S. aureus* as a clone with impaired catalase and host adaptation. This species illustrates that specialization of pathogens is associated with gene loss, not with gene gain.
